# Clinical Outcome of Septic Patients With Heart Failure With Preserved Ejection Fraction Presenting to the Emergency Department of a Tertiary Hospital: A Retrospective Cohort Study

**DOI:** 10.3389/fmed.2020.517999

**Published:** 2020-10-30

**Authors:** Ralphe Bou Chebl, Iskandar Berbari, Rawan Safa, Ali Shami, Mohammad Sabra, Sarah Jamali, Maha Makki, Hani Tamim, Gilbert Abou Dagher

**Affiliations:** Department of Emergency Medicine, American University of Beirut Medical Center, Beiru, Lebanon

**Keywords:** heart failure, diastolic, sepsis, mortality, emergency department

## Abstract

**Background:** Patients with heart failure with preserved ejection fraction (HFpEF) may be at a higher risk of mortality from sepsis than patients without heart failure.

**Objective:** The aim of this study is to compare sepsis-related morbidity and mortality between patients with HFpEF and patients without heart failure presenting to the emergency department (ED) of a tertiary medical center.

**Design:** Single-center retrospective cohort study conducted at an academic ED between January 1, 2015 and December 31, 2018.

**Patients:** Patients with a diagnosis of sepsis were included.

**Main Measures:** Bivariate and multivariate analyses were performed to look at differences in demographics, infection, and treatment parameters as well as outcomes of patients with sepsis. The primary outcome of the study was in-hospital mortality. Secondary outcomes included ED mortality, lengths of stay, and treatment differences between both groups.

**Key Results:** A total of 1,092 patients presented with sepsis to the ED, of which 305 (27.93%) had HFpEF. There was no significant difference in in-hospital mortality between the two groups (40.7% vs. 37.4%; *p* = 0.314). However, there was a significant increase in ED mortality for septic HFpEF patients compared to non-heart failure patients (2.4 vs. 0.4%; *p* = 0.003). Septic HFpEF patients presenting to the ED were older than non-heart failure patients (76.84 vs. 68.44 years old; *p* < 0.0001). On the other hand, there was no significant increase in the use of vasopressors in the first 24 h between both groups. There was a significantly higher rate of intubation in the first 48 h for septic HFpEF patients (17.5 vs. 8.9%; *p* < 0.0001). Finally, there was significantly less intravenous fluid requirement at 6 h (1.94 L vs. 2.41L; *p* < 0.0001) and 24 h (3.11 L vs. 3.54L; *p* = 0.004) for septic patients with HFpEF compared to non-heart failure patients.

**Conclusion:** Septic HFpEF patients experienced an increase in ED mortality, intubation, and steroid use compared to septic non-heart failure patients.

## Introduction

Heart failure and sepsis are major public health problems. More than 5.8 million in the United States and 23 million people worldwide have heart failure (1). The prevalence of heart failure with preserved ejection fraction (HFpEF) relative to heart failure with reduced ejection fraction (HFrEF) is rising at an alarming rate of 1% per year, with 50% of heart failure patients currently having HFpEF (2, 3). Morbidity, mortality, and healthcare costs of HFpEF are on par with HFrEF (4). Sepsis, on the other hand, affects more than 600,000 patients in the USA each year and is associated with high mortality: up to 70% in seriously ill patients (5–7). It is a condition that is caused by the host's response to a bacterial infection. The cytokines released affect multiple organs, including the cardiovascular system (6). Sepsis has been shown to cause myocardial suppression, as well as diastolic dysfunction and decreased cardiac index (8). HFpEF patients may be at a higher risk of mortality from sepsis than patients without heart failure due to insufficient cardiovascular reserves during systemic infection (8). There is limited data available on the association between heart failure with preserved Ejection Fraction with the clinical outcome and mortality rates in sepsis and septic shock (9). The aim of this study is to compare sepsis-related morbidity and mortality between patients with HFpEF and patients without heart failure presenting to a tertiary medical center.

## Methods

### Study Design and Setting

This was a single-center, retrospective, cohort study conducted in an academic emergency department (ED) between January 1, 2015, and December 31, 2018. The hospital's electronic system was used to query patients' charts. All demographic information, including comorbidities, vital signs, laboratory results, and resuscitation parameters, were extracted from scanned charts by research fellows. Multiple meetings with the principal investigators were conducted to standardize the data extraction and entry process. This study was approved by the Institutional Review Board of the American University of Beirut and the hospital's IRB (BIO-2018-0106) and carried out in accordance with the recommendations provided. The research was performed according to ethical principles and in compliance with all prevailing and applicable laws, rules, and regulations and policies regarding the protection of human subjects and research conduct as outlined by the declaration of Helsinki. Subject privacy and data confidentiality were of paramount concern at all times, and every effort was made to protect subjects' rights and welfare.

### Patient Selection

Patients' ED encounters were filtered by an experienced data user using the hospital's EHR (electronic health records) via an extensive structured keyword search and ICD-9 coding (International Statistical Classification of Diseases, Ninth Revision and Related Health Problems). The ICD-9 diagnoses retrieved were sepsis (995.91) and septic shock (785.52). A list of all patients who were diagnosed with sepsis or septic shock between January 1, 2015, and December 31, 2018, was therefore compiled (IRB #BIO-2018-0106). Sepsis was defined according to the sepsis-3 definition as the presence of an infection with signs of organ dysfunction, which were represented by an increase in the Sequential [Sepsis-related] Organ Failure Assessment (SOFA) score of two points or more. Septic shock was defined by a vasopressor requirement to maintain a mean arterial pressure of 65 mm Hg and a serum lactate level >2 mmol/L (>18 mg/dL) in the absence of hypovolemia (10). Patients younger than 18 years, cardiac arrest patients, patients transferred from another hospital, trauma patients, pregnant patients, and HFrEF patients as well as patients who did not have an echocardiogram were excluded from the study. We elected to exclude patients with a reduced ejection fraction as these patients usually have high left ventricular (LV) filling pressures and often have diastolic dysfunction, and our aim was to compare patients with HFpEF to patients with a normal cardiac function and echo.

### Exposure: Heart Failure With Preserved Ejection Fraction

Patients who were included in the study were stratified according to the presence of HFpEF. HFpEF was identified via the revision of echocardiography reports performed by cardiologists at our institution. The echocardiography report was considered valid if the procedure was performed at most 1 month before the admission date. Heart failure with preserved ejection fraction was defined according to the recommendation given by the American Society of Echocardiography (ASE) and the European Association of Cardiovascular Imaging (EACVI) (11). In order for patients to meet the definition, they had to meet three criteria. First, signs and/or symptoms of heart failure had to be present (11). Second, normal systolic LV function (LV ejection fraction > 50 %) should be present on echocardiography (11). Third, evidence of LV diastolic dysfunction had to also be present. According to the ASE/EACVI guidelines, diastolic dysfunction was based on four variables: Septal e′ < 7 cm/s or Lateral e′ < 10 cm/s, an average E/e′ > 14, left atrium (LA) volume index > 34 mL/m^2^ and peak tricuspid regurgitation (TR) velocity > 2.8 m/s (11). All these variables are routinely reported in echocardiograms at our institution.

### Outcome Measures

The aim was to characterize the presentation of sepsis among HFpEF patients compared to those without heart failure and assess associated clinical outcomes. The primary outcome of the study was in-hospital mortality. Secondary outcomes included ED mortality, lengths of stay, and differences in treatment interventions between both groups. The infection site was determined from documentation in the medical record, culture results (blood, sputum, urine, other fluids), and/or radiology reports (such as chest X-rays). Laboratory results were obtained from the hospital's EHR system. Information about antibiotics and vasopressors, time to their initiation, and duration of their use was obtained by reviewing the scanned ED order sheet. Disposition status was also recorded by reviewing admission and discharge documents.

### Statistical Analysis

Statistical Package for Social Sciences (SPSS) version 25.0 (Armonk, New York, USA: IBM Corp) was used to perform all statistical analyses. Continuous and categorical variables were presented as mean ± SD and frequency/percentages, respectively. The Shapiro–Wilk test, Kurtosis and Skewness Z-score, and visualization of histograms were used to check for normality of distribution of all continuous variables. Time to antibiotics was presented as median and interquartile range (IQR). The different parameters were then stratified by whether or not patients had HFpEF (HFpEF only or no HFpEF and no HFrEF). Pearson's χ2 test was used to assess for statistical significance for the categorical variables, while the Student's *t*-test and the Mann–Whitney *U-*test were used for the continuous ones. Tests were interpreted at a significance level alpha = 0.05. Multivariable analyses were performed to ascertain the association between HFpEF status and mortality [one for in-hospital mortality (Table 5) and another for ED mortality (Table 6)] in the septic population via a logistic regression. Another multivariate analysis was performed to determine the association between total LOS of in-hospital survivors and HFpEF via a linear regression (Table 7). A backward selection procedure, with the significance level for variable removal from the model set at 0.05, was conducted. All clinically and statistically significant variables in the bivariate analysis level in were included in the multivariate analysis. The variables included in the model are listed in each table. The results were described as odd ratios (ORs) and their corresponding 95% CI.

## Results

### Patient Characteristics at Presentation to the ED

One thousand ninety-two septic patients were included in this study, of which 305 (27.93%) had HFpEF on admission. On the other hand, 787 patients (72.07%) had neither HFpEF nor HFrEF. There was an equal gender distribution among patients with HFpEF (50.5 vs. 49.5%; *p* = 0.285). Patients with HFpEF were significantly older than non-heart failure patients (76.84 vs. 68.44 years old; *p* < 0.0001). The HFpEF group had a significantly higher rate of chronic kidney disease (21.9 vs. 12.4%; *p* < 0.0001), hypertension (71.5 vs. 59.6%; *p* < 0.0001), dyslipidemia (37.7 vs. 26.9%; *p* < 0.0001), coronary artery disease (36.7 vs. 24.0%; *p* < 0.0001), atrial fibrillation (24.9 vs. 17.0%; *p* = 0.003), diabetes mellitus (44.6 vs. 31.9%; *p* < 0.0001), and chronic obstructive pulmonary disease (COPD) (13.1 vs. 9.0 %; *p* = 0.045). Table 1 summarizes the patient's comorbidities. The most common symptom for HFpEF patients was dyspnea on exertion, which was seen in 80% of patients, followed by orthopnea and paroxysmal nocturnal dyspnea which were seen in 65 and 35% of patients. As for the signs, 85% of patients were found to have pitting edema, 75% were found to have crackles on auscultation.

**Table 1:** Patients characteristics at presentation.

		**HFpEF only (*N* = 305)**	**No HFpEF and no HFrEF** **(*N* = 787)**	
		**Mean** **±** **SD**		***p-value***
**Age (years)**		76.8 ± 10.9	68.4 ± 17.1	<0.0001
Vital signs	SBP (mmHg)	120.4 ± 28.6	115.0 ± 28.6	0.005
	DBP (mmHg)	62.1 ± 18.1	63.9 ± 18.0	0.135
	HR (bpm)	98.1± 23.6	105.1 ± 25.4	<0.0001
	Oxygen saturation (%)	92.6 ± 9.0	94.2 ± 7.2	0.004
	Temperature (°C)	37.4 ± 1.1	37.4 ± 1.2	0.826
	Respiratory rate (Bpm)	22.9 ± 6.1	22.2 ± 6.5	0.127
		***N*** **(%)**		***p-value***
Gender	Female	154 (50.5%)	369 (46.9%)	0.285
Smoking		49 (23.1)	77 (12.3)	<0.0001
PMH	Chronic kidney disease	66 (21.9)	95 (12.4)	<0.0001
	End stage renal disease	19 (6.2)	45 (5.7)	0.747
	Hypertension	218 (71.5)	469 (59.6)	<0.0001
	Dyslipidemia	115 (37.7)	212 (26.9)	<0.0001
	Coronary artery disease	112 (36.7)	189 (24.0)	<0.0001
	Atrial fibrillation	76 (24.9)	134 (17.0)	0.003
	TIA or stroke	23 (7.6)	45 (5.7)	0.258
	Vascular disease	21 (6.9)	34 (4.3)	0.082
	Diabetes mellitus	136 (44.6)	251 (31.9)	<0.0001
	COPD	40 (13.1)	71 (9.0)	0.045
	History of cancer	107 (35.1)	320 (40.7)	0.090
	Currently in remission	14 (17.7)	42 (19.3)	0.764
	Currently on treatment	53 (59.6)	146 (59.8)	0.963

*°C, degrees Celsius; bpm, beats per minute; bpm, beats per minute; bpm, breath per minute; Bpm, breaths per minute; COPD, chronic obstructive pulmonary disease; DBP, diastolic blood pressure; HFpEF, heart failure with preserved ejection fraction; HFrEF, heart failure with reduced ejection fraction; HR, heart rate; mmHg, millimeter of mercury; PMH, past medical history; SBP, systolic blood pressure; SD, standard deviation*.

### Vital Signs and Laboratory Tests

On presentation to the ED, HFpEF patients had a significantly higher systolic blood pressure (120.47 vs. 114.99 mmHg; *p* = 0.005), a significantly lower heart rate (98.26 vs. 105.11 bpm; *p* < 0.0001), and a significantly lower oxygen saturation (92.57 vs. 94.23%; *p* = 0.004). The rest of the vital signs at presentation were similar between the two cohorts (Table 1).

As for the laboratory studies at presentation, the two groups had similar lactate, albumin, hemoglobin, hematocrit, BUN, creatinine, sodium, absolute neutrophil count, lymphocyte count, white blood cell count, chloride, total bilirubin, troponin, potassium, magnesium, calcium, procalcitonin, pH, bicarbonate, PaCO_2_, PT, PTT, and INR. However, patients with HFpEF had a significantly lower phosphate level (3.44 vs. 3.69 mg/dL; *p* = 0.022) (Table 2). The median value for pro-BNP for HFpEF patients was 955 with an IQR of 1,585.

**Table 2:** Laboratory results at presentation.

	**HFpEF only** **(*N* = 305)**	**No HFpEF and no HFrEF** **(*N* = 787)**	
	**Mean** **±** **SD**		***p-value***
Lactate at presentation mmol/L	3.5 ± 2.7	3.7 ± 3.0	0.444
Lactate second variable mmol/L	3.1 ± 3.9	3.2 ± 3.4	0.781
Albumin at presentation g/L	28.0 ± 6.5	27.6 ± 6.8	0.411
Procalcitonin ng/ml	9.6 ± 20.1	14.2 ±29.6	0.079
Glucose mg/dL	156.4 ± 76.3	151.8 ± 87.0	0.471
Hemoglobin g/dL	10.9 ± 1.9	11.0 ± 2.3	0.498
Hematocrit %	32.9 ± 6.2	33.0 ± 7.0	0.883
BUN mg/dl	38.5 ± 30.6	35.5 ± 27.7	0.119
Creatinine mg/dl	1.8 ± 1.5	1.7 ± 1.8	0.667
Baseline creatinine (for patients with CKD) mg/dL	1.9 ± 0.6	1.8 ± 0.6	0.623
Sodium mmol/L	135.6 ± 6.5	135.3 ±6.5	0.496
Absolute neutrophil count /cu.mm	10,439.6 ± 7,650.1	10,615.4 ± 8,627.8	0.756
Lymphocyte count %	13.9 ± 17.3	12.4 ± 14.9	0.194
WBC/cu.mm	13,030.9± 9,163.8	13,362.4 ± 10,946.7	0.640
Bicarbonate mmol/L	21.9 ± 7.2	21.2 ± 5.6	0.105
Chloride mmol/L	97.1 ± 7.9	97.2 ± 7.1	0.763
Bilirubin total mg/dL	1.3 ± 2.9	1.6 ±3.1	0.319
Troponin ng/mL	0.1 ± 0.1	0.1 ± 0.2	0.676
Potassium mmol/L	4.6 ± 2.7	4.7 ± 6.2	0.712
Magnesium mg/dL	1.9 ± 0.4	2.2 ± 6.3	0.311
Calcium mg/dL	8.6 ± 0.9	8.6 ± 1.1	0.932
Phosphate mg/dL	3.4 ± 1.5	3.7 ± 1.8	0.022
Ph (arterial)	7.36 ± 0.12	7.34 ± 0.11	0.098
PaCO_2_ mmHg	38.3 ± 20.2	35.9 ± 14.2	0.175
PT sec	18.69 ± 11.77	20.49 ± 16.25	0.185
PTT sec	33.88 ± 16.58	34.66 ±19.48	0.653
INR	1.6 ± 1.0	1.9 ± 2.6	0.259

*/cu.mm, per cubic millimeter; CKD, chronic kidney disease; g/dL, grams per deciliter; g/L, grams per liter; HFpEF, heart failure with preserved ejection fraction; HFrEF, heart failure with reduced ejection fraction; INR, International Normalized Ratio; mg/dL, milligrams per deciliter; mmHg, millimeter of mercury; mmol/L, millimoles per liter; ng/ml, nanograms per liter; PaCO_2_, partial pressure of carbon dioxide; PT, prothrombin time; PTT, partial thromboplastin time; SD, standard deviation; sec, seconds; WBC, white blood cells*.

### Patient Hospital Course

HFpEF patients had a slight increase in in-hospital mortality (40.7 vs. 37.4%; *p* = 0.314); however, this difference was not statistically significant. Moreover, there was a significantly higher rate of ED mortality in the HFpEF cohort (2.4 vs. 0.4%; *p* = 0.003). During their hospital course, patients with HFpEF had a lower amount of fluid administered during the first 6 h (1.94 L vs. 2.41 L; *p* < 0.0001) and 24 h since ED admission (3.11 L vs. 3.54 L; *p* = 0.004) compared to patients without HFpEF. Among survivors, the hospital length of stay was significantly lower for patients with HFpEF compared to non-heart failure patients (257.78h vs. 317.36 h; *p*= 0.03). Over the first 24h, more HFpEF patients received steroids (23.4 vs. 18.1%; *p* = 0.048). Moreover, over the first 48 h, a higher number of HFpEF patients were intubated compared to patients without heart failure (17.5 vs. 8.9%; *p* < 0.0001) (Table 3).

**Table 3:** Patient hospital course.

			**HFpEF only** **(*N* = 305)**	**No HFpEF and no HFrEF** **(*N* = 787)**	
			**Mean** **±** **SD**		***p-value***
IV fluids (L)	IV fluid given during the first 6 h		1.94 ± 1.74	2.41 ± 1.82	<0.0001
	IV fluid given in the first 24 h		3.11 ± 2.23	3.54 ± 2.19	0.004
LOS	LOS in the ED (h)	Overall	15.77 ± 18.86	16.66 ± 21.93	0.533
		Survivors	15.46 ± 18.71	16.66 ± 21.94	0.40
		Non-survivors	29.16 ± 21.71	17.61 ± 22.68	0.47
	Total hospital LOS (h)	Overall	349.98 ± 391.15	392.64 ± 581.29	0.238
		Survivors	257.78 ± 256.94	317.36 ± 457.21	0.03
		Non-survivors	484.58 ± 500.76	518.87 ± 727.95	0.63
			***N*** **(%)**		***p-value***
Medication used	Norepinephrine given in the first 24 h		95 (31.1)	207 (26.3)	0.108
	Dopamine given in the first 24 h		7 (2.3)	9 (1.1)	0.155
	Epinephrine given in the first 24 h		6 (2.0)	22 (2.8)	0.437
	Dobutamine given in the first 24 h		2 (0.7)	7 (0.9)	1.00
	Steroids given in the first 24 h		71 (23.4)	142 (18.1)	0.048
Admission	Admitted to the GPU		201 (66.1)	503 (64.2)	0.561
	Admitted to the ICU		166 (54.6)	413 (52.7)	0.568
Intubation	Intubation in the first 24 h		58 (19.1)	118 (15.1)	0.104
	Intubation in the first 48 h		53 (17.5)	70 (8.9)	<0.0001
Mortality in the ED			7 (2.4)	3 (0.4)	0.003
In hospital mortality			124 (40.7)	294 (37.4)	0.314

*ED, emergency department; GPU, general practitioner unit; h: hours; HFpEF, heart failure with preserved ejection fraction; HFrEF, heart failure with reduced ejection fraction; ICU, intensive care unit; IV, intravenous; L, liters; LOS, length of stay; SD, standard deviation*.

### Infection Characteristics

There was no difference in the percentage of patients receiving antibiotics in the two groups. Time to antibiotic administration was also similar between the two cohorts (median: 2.1 h vs. 2.25 h; interquartile range and interquartile range (IQR) 2.85 h vs. 3.86 h). In both groups, the most common site of infection was the lungs. However, urinary tract infections were more common in HFpEF septic patients compared to septic patients without HFpEF (40.2 vs. 29.2%; *p* = 0.001). Moreover, HFpEF patients had less gastrointestinal infections when compared to the non-heart failure group (10.6 vs. 17.8%; *p* = 0.004). Bacteria were found mostly in blood in the two groups. However, there were more bacteria isolated from the urine in the HFpEF cohort (33.9 vs. 27.3%; *p* = 0.032). The most common bacteria found in blood, urine, and wound, in both groups, was *E. coli*. The most common bacteria found in sputum was Pseudomonas aeruginosa and Acinetobacter baumannii in the HFpEF group and *E. coli* in the non-heart failure group (Table 4).

**Table 4:** Infection characteristics.

			**HFpEF only (*N* = 305)**	**No HFpEF and no HFrEF** **(*N* = 787)**	***p-value***
Antibiotics	Time from ED admission to antibiotic initiation (h)	Median	2.1	2.25	0.864
		IQR	2.85	3.86	
			***N*** **(%)**		***p-value***
Antibiotics	Antibiotic used		294 (96.7)	752 (95.8)	0.487
	Appropriate choice of antibiotic		241 (92.7)	648 (93.5)	0.655
Site of infection	Lung		140 (46.7)	304 (39.3)	0.027
	Urine		121 (40.2)	228 (29.2)	0.001
	Gastrointestinal system		32 (10.6)	139 (17.8)	0.004
	Surgical site		5 (1.7)	14 (1.8)	0.879
	Skin		17 (5.7)	35 (4.5)	0.423
	Bone		2 (0.7)	2 (0.3)	0.323
	Peritoneum		3 (1.0)	7 (0.9)	1.00
	Liver		2 (0.7)	5 (0.6)	1.00
	Heart		7 (2.3)	16 (2.1)	0.776
	Gall bladder		7 (2.3)	19 (2.4)	0.913
	Intravascular catheter		2 (0.7)	8 (1.0)	0.735
Bacteria	Blood		137 (45.2)	362 (46.3)	0.749
	Urine		103 (33.9)	214 (27.3)	0.032
	Sputum		37 (12.3)	83 (10.7)	0.474
	Wound		21 (7.0)	41 (5.3)	0.269
Bacteria in blood	Staphylococcus coagulase negative		32 (10.5)	93 (11.8)	0.537
	*E. coli*		59 (19.3)	143 (18.2)	0.654
	*Streptococcus*		12 (3.9)	29 (3.7)	0.846
Bacteria in urine	*Acinetobacter baumannii*		4 (1.3)	6 (0.8)	0.479
	*E. coli*		64 (21.0)	145 (18.4)	0.335
	*Klebsiella pneumonia*		18 (5.9)	35 (4.4)	0.316
Bacteria in sputum	*Pseudomonas*		9 (3.0)	15 (1.9)	0.291
	*E. coli*		8 (2.6)	17 (2.2)	0.646
	*Acinetobacter baumannii*		9 (3.0)	13 (1.7)	0.170
Bacteria in wound	*Staphylococcus coagulase negative*		4 (1.3)	5 (0.6)	0.276
	*Pseudomonas*		3 (1.0)	5 (0.6)	0.693
	*Enterococcus*		6 (2.0)	3 (0.4)	0.017
	*E. coli*		9 (3.0)	17 (2.2)	0.442

*ED, emergency department, E. coli, Escherichia coli; h, hours; HFpEF, heart failure with preserved ejection, HFrEF, heart failure with reduced ejection fraction; IQR, interquartile range; SD, standard deviation*.

### Multivariate analysis

Multivariate analyses were conducted to determine the association between HFpEF and hospital mortality, HFpEF and ED mortality, and HFpEF and total LOS of in-hospital survivors, while taking into consideration all statistically and clinically relevant variables in the bivariate analysis (Tables 5–7).

**Table 5:** Multivariate logistic regression of potential predictors of Hospital mortality.

**Variables**	**OR (95 % CI)**	***p-value***
**Hospital mortality (reference: no)**		
HFpEF only	1.21 (0.90–1.62)	0.21
Lactate at presentation	1.10 (1.03–1.18)	0.004
Intubation first 24 h	2.53 (1.74–3.69)	<0.0001
Site of infection—lung	1.78 (1.36–2.34)	<0.0001

*Variables included in the model were*.

*Imposed: HFpEF - (reference: no HFpEF and no HFrEF)*.

*Stepwise: age; gender (reference: female); vital signs heart rate (HR); smoking; chronic kidney disease; hypertension; dyslipidemia; coronary artery disease; atrial fibrillation; diabetes mellitus; chronic obstructive pulmonary disease (COPD); lactate at presentation; procalcitonin; creatinine; phosphate; IV fluid first 6 h; IV fluid given in the first 24 h; norepinephrine given in the first 24 h; steroids given in the first 24 h; intubation first 24h; intubation in the first 48 h; site of infection (lung; urine; gastrointestinal system), urine bacteria; bacteria in wound—Enterococcus*.

*CI, confidence interval; h, hours; HFpEF, heart failure with preserved ejection fraction; HFrEF, heart failure with reduced ejection fraction; IV, intravenous; OR, odds ratio*.

**Table 6:** Multivariate logistic regression of potential predictors of Mortality in the ED.

**Variables**	**OR (95 % CI)**	***p-value***
**ED mortality (reference: no)**		
HFpEF only	9.07 (1.98–41.60)	0.005
Vital signs HR(increase by 10 units)	1.42 (1.06–1.89)	0.02
Intubation first 24 h	23.45 (4.61–119.25)	<0.0001
Site of infection—lung	0.11 (0.02–0.66)	0.02
Bacteria in wound—Enterococcus	58.94 (6.01–578.23)	<0.0001

*Variables included in the model were*.

*Imposed: HFpEF—(reference: no HFpEF and no HFrEF)*.

*Stepwise: age; gender (reference: female); vital signs heart rate (HR); smoking; chronic kidney disease; hypertension; dyslipidemia; coronary artery disease; atrial fibrillation; diabetes mellitus; chronic obstructive pulmonary disease (COPD); lactate at presentation; procalcitonin; creatinine; phosphate; IV fluid first 6 h; IV fluid given in the first 24 h; norepinephrine given in the first 24h; steroids given in the first 24 h; intubation first 24 h; intubation in the first 48 h; site of infection (lung; urine; gastrointestinal system), urine bacteria; bacteria in wound—Enterococcus*.

*CI, confidence interval; ED, emergency department; h, hours; HFpEF, heart failure with preserved ejection fraction; HFrEF, heart failure with reduced ejection fraction; IV, intravenous; OR, odds ratio*.

**Table 7:** Multivariate linear regression of potential predictors of LOS of the survivors in hospital.

**Variables**	****β** (95 % CI)**	***p-value***
**LOS**		
HFpEF only	−42.20 (−111.77; 27.37)	0.23
Vital signs HR(increase by 10 units)	−14.57 (−27.82; −1.31)	0.03
History of cancer	89.62 (23.90; 155.34)	0.008
IV fluid given in the first 24 h	21.37 (7.12; 35.63)	0.003

*Variables included in the model were*.

*Imposed: HFpEF—(reference: no HFpEF and no HFrEF)*.

*Stepwise: age; gender (reference: female); vital signs heart rate (HR); smoking; chronic kidney disease; hypertension; dyslipidemia; end–stage renal disease; TIA or stroke; vascular disease; history of cancer; coronary artery disease; atrial fibrillation; diabetes mellitus; COPD; lactate at presentation; procalcitonin; creatinine; phosphate; IV fluid first 6 h; IV fluid given in the first 24 h; norepinephrine given in the first 24 h; steroids given in the first 24 h; intubation first 24 h; intubation in the first 48 h; site of infection (lung; urine; gastrointestinal system), urine bacteria; bacteria in wound—Enterococcus*.

*CI, confidence interval; h, hours; HFpEF, heart failure with preserved ejection fraction; HFrEF, heart failure with reduced ejection fraction; IV, intravenous*.

There was no significant difference in in-hospital mortality between patients with HFpEF and patients without heart failure (OR 1.21, 95% CI 0.90–1.62) after adjusting for confounders. Patients who underwent intubation in the first 24 h (OR 2.53, 95% CI 1.74–3.69) and patients whose lungs were the site of infection (OR 1.78, 95% CI 1.36–2.34) had higher odds of in-hospital mortality. Moreover, for every unit increase of lactic acid at presentation (OR 1.10, 95% CI 1.03–1.18), patients had higher odds of in-hospital mortality (Table 5). Patients with HFpEF had significantly higher odds of ED mortality compared to patients without heart failure (OR 9.07, 95% CI 1.98–41.60) after adjusting for confounders (Table 6). After adjusting for comorbidities and other confounders, there was no significant difference in total LOS of in-hospital survivors with HFpEF compared to in-hospital survivors without heart failure (β −42.20, 95% CI −111.77–27.27) (Table 7).

## Discussion

Our results showed that HFpEF patients had a slightly increased in-hospital mortality as compared to the non-heart failure group; however, this difference was not statistically significant. On the other hand, septic HFpEF patients had a significantly higher ED mortality compared to septic patients without heart failure. Moreover, HFpEF patients were found to have 1.21 times the odds of hospital mortality compared to controls and 9 times the odds for ED mortality after adjusting for potential confounders. These results are in line with the literature, where a meta-analysis by Sanfilippo et al. found that septic patients with diastolic dysfunction had a significantly higher mortality rate than patients with no diastolic dysfunction (9). There are several proposed reasons for the increased mortality in HFpEF patients. First, the cytokines released in sepsis lead to worsening diastolic dysfunction and myocardial suppression, as well as profound vasoplegia and an increase in vascular permeability and capacitance (8, 9, 11). This hypovolemic state is also accompanied by a reflex tachycardia, which worsens the LV filling, mainly by decreasing the diastolic time (8, 9, 11). This cycle leads to a decreased cardiac output and poor perfusion which worsens organ failure. Landesberg et al. also conducted a retrospective study and found that diastolic dysfunction, in particular, when associated with a low cardiac output and stroke volume (SV) is a stronger predictor of early mortality in patients with sepsis and septic shock (6).

Second, sepsis also causes rhythm disturbances, and in a recent study, sepsis was an independent predictor of new-onset atrial fibrillation (AF) (12). AF is common in HFpEF as it is identified in two-thirds of HFpEF patients, and its presence is associated with increased morbidity and mortality (13). This was reflected in our study, where HFpEF septic patients had a significantly higher percentage of atrial fibrillation as compared to non-heart failure patients. Aggressive fluid resuscitation remains one of the mainstays of sepsis management. The surviving sepsis campaign advocates for 30 cc/kg of fluid as part of the initial treatment of sepsis (14–16). However, aggressive fluid resuscitation has fallen out of favor and can be particularly hazardous in heart failure patients with preserved ejection fraction (17–20). Excessive fluid loading to the non-compliant LV may aggravate lung congestion and cardiogenic pulmonary edema which is common in sepsis (21–23). In our study, even though HFpEF patients received less fluids than controls (1.94 L vs. 2.41 L; *p* < 0.0001), they had a higher rate of intubation, probably due to cardiogenic pulmonary edema. Due to the impaired LV relaxation and the sepsis-induced myocardial suppression, these patients are very sensitive to fluid and can develop pulmonary edema rapidly.

It is interesting to note that there was no difference in hospital lengths of stay between both groups nor were there any differences in the lactate acid level, the site of infections, or the most commonly isolated bacteria. One possible explanation for the length of stay similarity could be that HFpEF has a higher mortality early on in the course of sepsis, as witnessed by their high ED mortality in our cohort and their increased intubation rates. However, if they overcome this early period, HFpEF patients tend to have a similar hospital course like non-heart failure patients. Nonetheless, emergency physicians should be aware of their increased mortality. The authors recommend early aggressive measures and early coordination and consultation with cardiology specialists as well as critical care specialists when dealing with a septic HFpEF patient.

### Limitations

Given that this was a retrospective chart review cohort study, the most important limitation is the biases associated with this kind of study. In order to minimize those, frequent meetings were held between the principal investigator and research assistants to standardize the way in which data were collected, entered, and cleaned. This study is from a referral academic center ED that deals with regional complicated cases, which could explain the increased mortality seen in both cohorts and could limit the generalizability of the results. Second, the delay in antibiotic administration (median: 2.1 vs. 2.25 h; interquartile range and interquartile range (IQR): 2.85 vs. 3.86 h) might have led to the increased morality, as it has been shown in several studies (20, 24, 25). It is important to note that both cohorts were found to be unmatched in terms of comorbidities, making our results difficult to interpret. In an effort to correct for this, all statistically and clinically significant characteristics on bivariate analysis were controlled for in the multivariable analysis in order to minimize confounding variables. It is important to note that our secondary outcomes, with the exception of ED mortality and LOS among in-hospital survivors, are the result of univariate analysis and as such are subject to confounders. As such, our results should be interpreted with caution. Moreover, patients spent an extended period of time in the ED overall (15.77 ± 18.86 h for HFpEF patients and 16.66 ± 21.93 for patients without heart failure); this should be kept in mind as it can limit the generalizability of the results. Another limitation of the study is the exclusion of patients with HFrEF. We understand that this is a substantial subgroup of patients with heart failure, but we chose to exclude these patients because the aim was to look at septic HFpEF and study their outcome as the literature on this topic is scarce. Furthermore, we conducted a study looking at the mortality of HFrEF septic patients as compared to controls at our institution and we were able to show that HFrEF septic patients had 2.5 times the odds for in-hospital mortality than patients without heart failure (26). Finally, while our study looked at HFpEF patients, it is important to note that our control patients might have had sepsis-induced diastolic dysfunction without heart failure symptoms and this might have increased their mortality (27).

## Conclusion

Septic HFpEF patients experienced an increased rate of ED mortality, intubation, and steroid use compared to controls. This indicates increased vulnerability for this subcategory of patients. It is important to note that, because of the increased risk of lung congestion and cardiopulmonary edema in these patients, it is preferable to start vasopressors early on for hemodynamic support than to potentially overwhelm them with fluids. Septic HFpEF patients should benefit from a multidisciplinary approach in order to improve their outcome. This is of particular importance in the early phase of the disease given the increased risk of ED noted in our study. ED physicians should promptly identify these patients and aim for individualized and optimal care.

## Data Availability Statement

The datasets generated for this study are available on request to the corresponding author.

## Ethics Statement

The studies involving human participants were reviewed and approved by Institutional Review Board of the American University of Beirut. Written informed consent for participation was not required for this study in accordance with the national legislation and the institutional requirements.

## Author Contributions

RC and GD made substantial contributions to the conception and design of the study. RC, GD, IB, RS, AS, MS, MM, and HT were responsible for the acquisition of data, analysis, and interpretation of data. RC, GD, IB, SJ, and RS were involved in drafting the manuscript. GD, RC, IB, and RS were responsible for revising the manuscript critically for important intellectual content. RC and GD took responsibility for the paper as a whole. All authors contributed substantially to its revision and approved the submitted version.

## Conflict of Interest

The authors declare that the research was conducted in the absence of any commercial or financial relationships that could be construed as a potential conflict of interest.

## Abbreviations

cu. mm: per cubic millimeter
%: percentage
AF: atrial fibrillation
ASE: American Society of Echocardiography
Bpm: beats per minute
BUN: blood urea nitrogen
cc/kg: cubic centimeters per kilogram
CI: Confidence interval
CKD: chronic kidney disease
COPD: chronic obstructive pulmonary disease
DBP: diastolic blood pressure
EACVI: European Association of Cardiovascular Imaging
*E. coli*: Escherichia coli
ED: emergency department
EHR: electronic health records
ESRD: end stage renal disease
g/dL: gram per deciliter
g/L: grams per liter
GPU: general practitioner unit
H: hours
HFpEF: heart failure with preserved ejection fraction
HFrEF: heart failure with reduced ejection fraction
HR: heart rate
ICD: International Statistical Classification of Diseases
ICU: intensive care unit
INR: International Normalized Ratio
IQR: interquartile range
IRB: institutional review board
IV: intravenous
L: liter
LOS: length of stay
LV: left ventricular
mg/dL: milligrams per deciliter
mmHg: millimeter of mercury
mmol/L: millimoles per liter
ng/ml: nanogram per milliliter
NT-proBNP: NT-pro B-type natriuretic peptide
OR: odd ratio
PaCO_2_: partial pressure of carbon dioxide
PCWP: pulmonary capillary wedge pressure
pg/ml: picogram/milliliter
PT: prothrombin time
PTT: partial thromboplastin time
SBP: systolic blood pressure
SD: Standard deviation
Sec: second
SOFA: Sequential [Sepsis-related] Organ Failure Assessment
SPSS: Statistical Package for Social Sciences
SV: stroke volume
TIA: transient ischemic attack
USA: United states of America
χ2: Chi square test.
